# Educator beliefs and organizational constraints: Factors that influence informal education about fish consumption advisories in a southeastern US state

**DOI:** 10.1080/27658511.2023.2259716

**Published:** 2023-09-27

**Authors:** Kathleen M. Gray, Margaret R. Blanchard, Catherine E. LePrevost

**Affiliations:** a Institute for the Environment, University of North Carolina at Chapel Hill, Chapel Hill, NC, USA; b Department of STEM Education, North Carolina State University, Raleigh, NC, USA; c Department of Applied Ecology, North Carolina State University, Raleigh, NC, USA

**Keywords:** environmental health, informal science education, self-efficacy, fish consumption, beliefs, advisories

## Abstract

All US states, and many countries around the world, have waterways with environmental health advisories intended to protect individuals from harmful chemicals in fish, yet little is known about how informal science educators, even those who engage anglers along waterways, incorporate advisory information into their educational activities. This study, grounded in environmental health literacy, investigated the practices, knowledge, and beliefs of 24 informal educators housed in varied agencies and organizations in a southeastern US state. Participants described a range of educational activities and identified organizational constraints on their education about fish consumption advisories, which varied by organization type. Their knowledge of relevant environmental health concepts was incomplete, and they described health and teaching beliefs consistent with limited focus on advisory education. Local government and nonprofit educators were well positioned to educate anglers about advisories, due to their freedom to design and deliver instruction and their regular contact with anglers. Educators in wildlife agencies had more contact with anglers and were identified as potential conduits given their interactions, but organizational constraints (such as educators’ ability to choose content/pedagogy and conflicting missions of agencies) would need to be addressed.

## 1. Introduction

Annually, millions of people learn about environmental science in informal science education contexts, which can include museums, environmental education centers, and even participation in recreational activities, such as fishing (National Research Council [NRC], 2009). Interactions in these contexts may not have explicit goals related to teaching and learning science; instead learning may occur spontaneously and at opportune moments. Such is often the case when anglers learn about fish consumption advisories (FCAs), which inform people who eat fish how to avoid or reduce potential exposure to harmful chemicals in fish.

All 50 US states and some tribes and US territories issue FCAs, identifying fish species in specific bodies of water that have contamination levels that exceed health-based standards (U.S. Environmental Protection Agency [USEPA], n.d.). These advisories are typically issued by state and local health agencies rather than environmental agencies. The most common contaminants included in advisories are mercury and polychlorinated biphenyls (PCBs), which can cause serious health problems when consumed at high levels (Agency for Toxic Substances and Disease Registry [ATSDR], 2014; ATSDR, 2015). Those most vulnerable to adverse health effects include children, pregnant/breastfeeding women, subsistence anglers, and American Indian populations (Ha et al., 2017; Karagas et al., 2012; Moya, 2010).

Across the US, anglers have reported general awareness of FCAs in at least 10 studies (Niederdeppe et al., 2015); yet few studies have reported anglers using FCAs to inform decisions about whether to eat locally-caught fish (Tan et al., 2011). Unfortunately, the most vulnerable populations are often the least aware of these health advisories (He et al., 2021). Although men tend to be more aware of advisories than women, in a study in the southeastern US, anglers who shared their catch with women and children were least aware of FCA information (LePrevost et al., 2013). Lower FCA awareness, along with higher consumption rates, also was reported among socially and economically marginalized populations of anglers in multiple US regions (Johnson et al., 2016; Perez et al., 2012; Ratnapradipa et al., 2010).

Despite harmful environmental exposures occurring in communities across the US, education on contaminants, associated risks of exposure, and potential health effects is limited (Ramirez-Andreotta et al., 2016); and environmental health issues are not well represented in science education infrastructure. Similarly, information about educators who share environmental health information with lay publics is limited, though a few studies have focused on FCA education. Burger et al. (2003) identified classroom health care/nutrition programs as effective methods of educating pregnant and breastfeeding women about FCAs in the northeastern US. An educational intervention led by residents and housing/health professionals in a southeastern state reported reduced consumption of fish by pregnant women and children in a low-income, African-American community (Derrick et al., 2008). More recently, He et al. (2021) found that leveraging social networks was an effective way to engage hard-to-reach refugee populations who consume diets that are rich in fish and fish products. Other studies have focused on the credibility of those sharing information, with one study reporting that FCA information was viewed as more credible when shared by multiple agencies and people anglers frequently consulted for information (e.g. conservation officers, health professionals) (Jardine, 2003). Another study found that mistrust of government agencies among African American participants in the southeastern US was associated with skepticism of agency messaging on fish consumption (Driscoll et al., 2012).

Given that awareness of FCAs is low, especially among vulnerable populations, and the role of educators in sharing FCAs is not well understood, the purpose of this exploratory case study was to understand factors that influence how informal educators share FCA information with anglers. This study was grounded in environmental health literacy (EHL), a framework that describes a range of knowledge and skills that enable people to make health-protective decisions using available environmental data (Finn & O’Fallon, 2017). Representations of EHL tend to start with individual understanding of specific environmental risks and then lead to broader understanding, including strategies that empower people to reduce or eliminate environmental exposures that can harm health (Gray, 2018). EHL posits that environmental health knowledge, combined with information-seeking and decision-making skills and self-efficacy for specific behaviors, can inform collective action and community change. The emphasis on self-efficacy and collective action within EHL is not well developed but aligns with place-based education initiatives, especially those that explicitly incorporate issues of power and seek to foster environmental citizenship (Schild, 2016). Further, the EHL framework is relevant for communities experiencing environmental contamination because behavior change is typically a focus of efforts to eliminate harmful environmental exposures in such communities (Ramirez-Andreotta et al., 2016).

To date, efforts to measure the core constructs of EHL have tended to focus on knowledge acquisition rather than self-efficacy, skills, and behaviors (Gray & Lindsey, 2019), resulting in gaps in understanding how to operationalize this framework. Behavioral theories that identify efficacy beliefs as facilitators of action, such as social cognitive theory (SCT) (Bandura, 1998), provide insight that could inform EHL conceptualization and assessment. SCT asserts that we use existing knowledge and beliefs to interpret external situations and develop expectations that influence future behavior. Riggs and Enochs (1990) applied SCT to science teaching, developing a validated instrument to assess elementary teachers’ beliefs about science teaching and learning. Similar instruments have been used to assess the self-efficacy of informal educators (LePrevost et al., 2013). Specific to health behaviors, the Health Belief Model (HBM) (Rosenstock et al., 1988) identifies several core beliefs that facilitate health behaviors, including beliefs about: the extent of risk associated with an action (*susceptibility*); how severe negative health consequences of an action may be (*severity*); the *benefits* of taking action; *barriers* to taking action; and *self-efficacy* (Orji et al., 2012). The HBM has informed research on lay health advisors’ delivery of pesticide education to Spanish-speaking families (Quandt et al., 2013) and subsistence anglers’ perceptions about fish consumption (Driscoll et al., 2012).

Although several studies have provided insight into efficacy and normative beliefs of anglers (Niederdeppe et al., 2015, Tan et al., 2011; Teisl et al., 2011), limited information is available on the beliefs and practices of informal educators who share information with anglers, including what information they share, how they do it, and their associated efficacy beliefs. Employing the EHL framework provides an opportunity to examine how educators’ environmental health knowledge, teaching self-efficacy beliefs, and health beliefs may influence their educational practice. Thus, this exploratory case study addressed the following research questions: (1) How did participants describe their educational activities and teaching of advisory information to anglers, their families, and other public audiences? (2) How did they describe their environmental health knowledge and teaching efficacy beliefs (core components of EHL) related to fishing and FCAs? and (3) How did they describe their health beliefs (potential modifiers of health behaviors)?

## 2. Methods

This exploratory case study was conducted in a southeastern US state with a large population of licensed anglers, a statewide mercury advisory, and site-specific FCAs for PCBs and dioxins. The study employed concurrent mixed methods (Creswell & Plano Clark, 2007) to understand the environmental health knowledge, teaching efficacy and health beliefs, and educational practices of informal educators who teach anglers about FCAs. The population of educators who share FCA information in this state was unknown but estimated to be less than 80 people. To recruit participants, a list of educators engaged in FCA development and communication was created by the authors, two of whom have worked on FCA issues in the southeast for over a decade. The initial list included individuals and organizations who were required to educate about FCAs and was expanded through snowball sampling (Noy, 2009). Potential participants were recruited by phone, email, and in-person gatherings of relevant organizations. All study protocols received Institutional Review Board approval (#6263).

Twenty-four informal educators and environmental/public health professionals consented to participate in the study. They identified as male (*n* = 13), female (*n* = 10), and other (*n* = 1); they also identified as White (*n* = 21), Latino (*n* = 2), other (*n* = 1), or did not respond (*n* = 2). Their ages ranged from 27 to 64 years, with an average age of 43. All participants had some education beyond high school, including an associate’s degree (*n* = 1), bachelor’s degrees (*n* = 6), master’s degrees (*n* = 16), and a professional degree (*n* = 1). The organizations represented by participants included environmental nonprofits (*n* = 8), state environmental/health/wildlife agencies (*n* = 8), local governments (*n* = 4), and land grant universities (*n* = 4).

### 2.1. Data collection and analysis

Table 1 presents an overview of core constructs and data collection instruments, which are described in more detail below.

#### 2.1.1. Interviews

Participants’ environmental health knowledge, teaching practices, and teaching and health beliefs were explored in interviews, which lasted between 45–90 minutes. Most interviews were conducted in person, in mutually agreed-upon locations, which included private offices at participants’ places of employment and private spaces in other locations (such as libraries). Three were conducted by phone. All interviews were audiotaped and transcribed verbatim.

The interview guide was designed to capture information related to participants’ environmental health knowledge, teaching and health beliefs, and educational practices. Original items were combined with questions excerpted and adapted from the Teacher Belief Interview (TBI; Luft & Roehrig, 2007). Items related to educational practices included the following: *How do you educate fishermen about fishing? What do you teach? Do you educate fishermen about FCAs? If not, why not?* Items designed to capture environmental health knowledge included the following: *What, if anything, do you tell people regarding how they can tell if fish are safe to eat? What are the two most important ideas or concepts for fishermen to understand related to FCAs?* Two TBI questions were adapted and incorporated into the interviews to examine teaching beliefs: *How do you decide what information to share? How do you know when fishermen understand?* Items also related to participants’ beliefs about the importance of FCAs and the likelihood that people would act on information shared by educators: *How important is it that fishermen know about FCAs? How likely is it that people would take action based on the information you might share about FCAs?*

The resulting transcripts consisted of 406 double-spaced pages, which were coded using *a priori* and emergent codes (Elliott, 2018). Approximately 20% of the data was independently co-coded by the first author and other trained coders, with > 80% interrater reliability in initial coding. For every response for which the coders did not initially agree, a negotiated code was determined through discussion and further review of interview transcripts (Patton, 2002). Given this agreement, the first author coded the rest of the transcripts using the coding protocol and reviewed them with co-coders, resulting in a codebook with six code families and 30 unique codes. Codes families focused on educational activities, environmental health knowledge, teaching efficacy beliefs, HBM concepts, information sources, and professional preparation. To further explore participants’ environmental health knowledge, all coded text was reviewed for comments related to a set of core environmental health concepts: hazard, dose, health effects, and vulnerable populations.^1^ Comments about ecosystem dynamics relevant to environmental exposure (e.g. size or age of fish) also were coded.

#### 2.1.2. Surveys

After each interview, participants completed the online surveys described below.

##### 2.1.2.1. Science teaching efficacy belief instrument (STEBI) (Riggs & Enochs, 1990).

To assess participants’ science teaching efficacy beliefs—both their personal science teaching efficacy and science teaching outcome expectancy associated with teaching about fish consumption—the STEBI was modified and administered as a 10-item quantitative questionnaire. Item wording was modified for informal science education contexts in which educators share information with fishermen, and the 1 to 5 scoring scale (for strongly disagree to strongly agree, respectively) was reversed for some items, using the language of the original instrument. Example items included: (a) *I typically am able to answer fishermen’s questions about the potential harms of eating contaminated fish* and (b) *Even educators with good teaching abilities cannot help some fishermen learn about the potential harms of eating contaminated fish* (reverse coded). Self-efficacy scores based on STEBI items and correct responses to the knowledge survey were calculated and compared to qualitative findings.

##### 2.1.2.2. FCA knowledge and beliefs survey.

An 8-item, selected- and constructed-response survey was developed to assess participants’ knowledge of FCA-related issues, such as the agencies that create FCAs, common contaminants, health effects of these contaminants, and advisories in their local area and the state. Several questions addressed core constructs of the Health Belief Model (e.g. susceptibility and severity), using Likert scale items.

##### 2.1.2.3. Demographic survey.

Participants also completed a demographic survey asking their name, gender, race, ethnicity, educational attainment, organization, and level of contact with fishermen.

## 3. Results

Findings are presented in two sections: (1) educational activities addressing FCAs and (2) environmental health knowledge and teaching and health beliefs.

### 3.1. Educational activities

**Figure 1.**
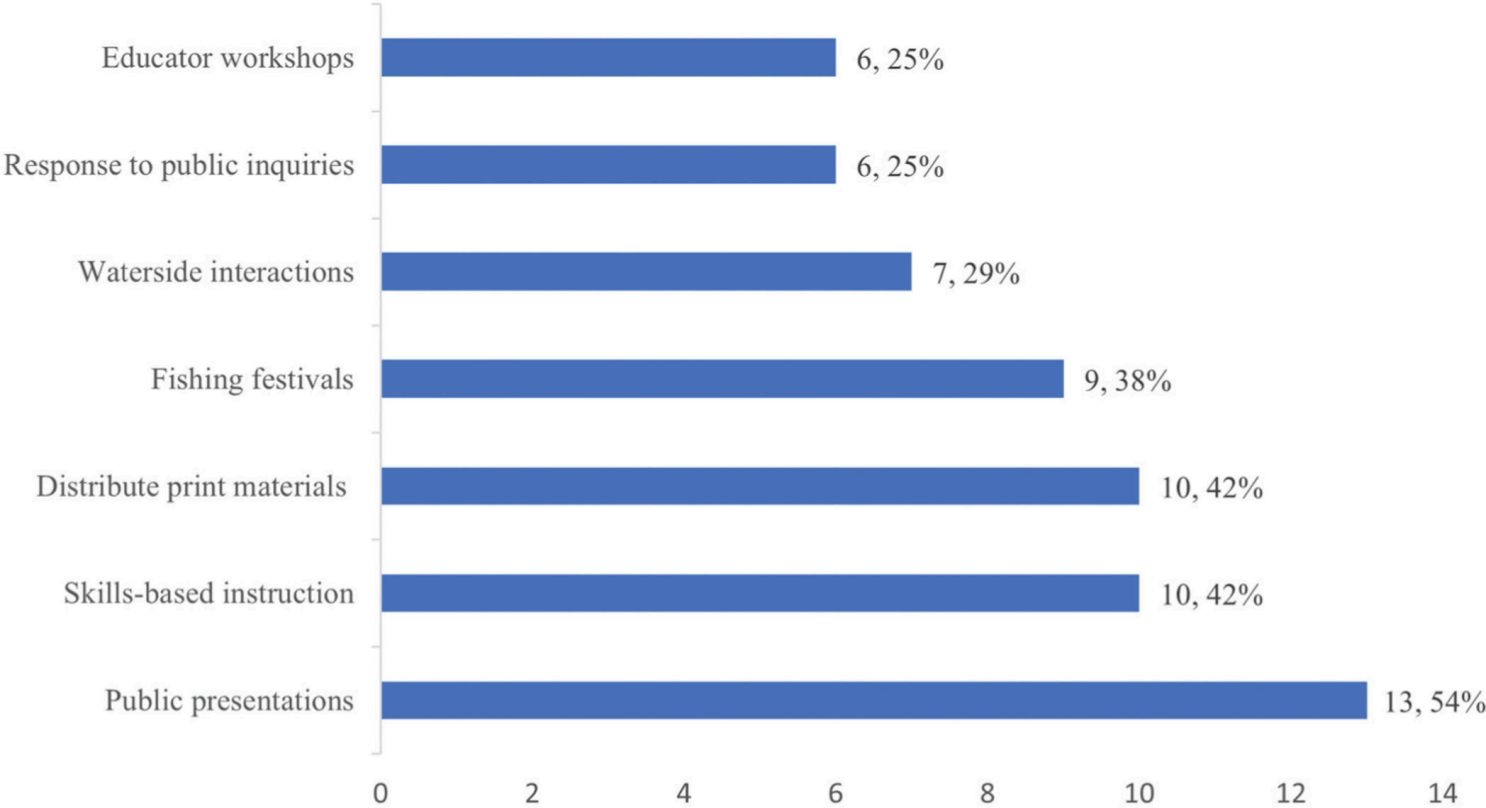
Common educational activities conducted by participants when working with anglers (N=24).

Participants shared information with anglers and lay audiences in varied ways, from public presentations on timely topics and skills-based instruction to conversations along waterways (Figure 1). All participants educated anglers about varied aspects of fishing, though none identified FCA education as a primary function of their jobs. They tended to address FCAs with anglers through brief, waterside conversations and responses to public inquiries; or they addressed FCAs briefly in longer interactions (e.g. workshops, public presentations). The subset who reported engaging the highest numbers of anglers annually were employed by the state wildlife agency, followed by local government staff and nonprofit educators.

Within organization types, the participants from local government environmental and recreation divisions (e.g. stormwater and parks departments) were most likely to regularly educate about FCAs (3 of 4; 75%), followed by nonprofit educators (5 of 8; 63%) and state agency personnel (e.g. environmental quality, health & human services) (2 of 8; 25%). Some participants reported engaging vulnerable populations in their educational activities, with 12 (50%) mentioning school-age children and seven (29%) mentioning subsistence anglers. A health department educator mentioned pregnant/breastfeeding women but only reported discussing FCAs with them if they asked questions about fish safety.

When participants were asked how they decided what information to share with anglers, eight (33%) indicated that they relied on curriculum guides, such as those created by Project WET, Project WILD, and health-based curricula. Five participants (from local government, nonprofit, and wildlife agencies) reported using requirements for scouting badges/patches in designing educational activities. Two local health department educators reported using established scripts to promote healthy behaviors. Only seven participants (29%) described having the freedom to develop new educational activities, based on their interests/expertise, the interests of anglers, instructional needs, or timely/locally relevant topics, such as industrial spills in local waterways.

#### 3.1.1. Confusion and constraints

In the southeastern state where the study was conducted, multiple state agencies and local governments shared information about FCAs with anglers. Some participants noted that this complexity contributed to confusion among anglers about whom to contact with questions. As a health agency educator (male, 2 years of professional experience) said, ‘Environmental health is structured differently in every county, so it just kind of depends on . . . who [the county] decides would be the best person for you to talk to’. Participants recognized that agency roles did not necessarily align with public perceptions. Although the wildlife agency was not responsible for issuing advisories nor educating about them, one participant (male, 3 yrs. exp.) asserted that anglers perceived this agency as ‘the authority on fish consumption’.

A number of participants described their FCA educational activities as being limited by their roles within an agency and what they believed they were allowed to address, in alignment with their stated missions. Specifically, about half (*n* = 11; 46%) identified institutional constraints as limiting their FCA education; seven of those participants were employed by state agencies. One state agency educator (male, 13 yrs. exp.) who was knowledgeable about FCAs said, ‘It’s not my job to communicate whether or not something is safe. . .I am not allowed to tell you [that]’. Similarly, a health agency educator (female, 3 yrs. exp.) indicated that she was allowed to educate about FCAs and health but could not discuss the origin of pollutants, saying ‘it’s in [another agency’s] jurisdiction to worry about source attribution and pollution’.

### 3.2. Environmental health knowledge and beliefs

In surveys, participants demonstrated knowledge of common contaminants included in FCAs in the state, with most accurately identifying mercury (state-wide advisory) and a majority accurately identifying polychlorinated biphenyls (PCBs) (Table 2). However, they were less familiar with health effects associated with these common FCA contaminants. Almost half identified neurological effects, and only a third identified cancer. Similarly, they were less familiar with the populations most vulnerable to harm from exposure to contaminants in fish. Almost half of participants correctly identified children as vulnerable, but fewer identified subsistence anglers and pregnant/breastfeeding women. Because advisories have the most stringent recommendations for children and women of childbearing age (including pregnant/breastfeeding mothers), it was expected that more participants would identify these populations as vulnerable. Finally, when asked to name the organization(s) that created fish consumption advisories in the state and given a list of answer choices, almost half correctly identified the state health agency.

#### 3.2.1. Core environmental health concept: Hazards

In interviews, most participants (*n* = 20; 83%) spoke about contaminants included in state-issued FCAs (i.e. dioxin, mercury, PCBs) without prompts. Eight (33%) also addressed safe handling procedures and spoilage, reporting that they relied on sensory cues associated with spoilage (e.g. bad smell) to determine whether fish were safe to eat. Several (*n* = 4; 17%) noted the limits of using sensory cues for FCA contaminants, because they have no discernible manifestations. An extension educator (female, 14 yrs. exp.) noted the challenges of talking to people about contamination that is not visible, saying:

How do you [talk about] things you can’t see? Like organic compounds, like PAH and PCBs, or mercury. There’s no way. You put two slices of tuna down here, I don’t know which one has high mercury and which one doesn’t.

When educating about FCAs, many participants (*n* = 17; 70%) explained that they addressed ecosystem concepts that related to environmental exposures, such as bioaccumulation of mercury/PCBs, and the terms *food chain*, *food web*, *bottom feeders*, and *top predators*. Four participants reported using these concepts to make information more understandable to anglers. A state agency educator (male, 13 yrs. exp.) said that he drew on anglers’ lived experience when educating about FCAs, saying ‘if you’re catching largemouth bass . . . or any top predator over a certain size . . . if you take it home and eat it, you’re pretty likely to be having something that’s over the state’s limit [for mercury]’.

#### 3.2.2. Core environmental health concept: Dose

About half of participants (*n* = 13; 54%) incorporated frequency of consumption or other concepts associated with dose and duration of exposure into their educational activities. A nonprofit program manager (female, 14 yrs. exp.) said her focus was, ‘to make sure they understand that there should be a limit’. A health agency educator (male, 3 yrs. exp.) noted that frequency of consumption affected health risks, saying, ‘on a one-off exposure, you’re just marginally increasing your risk’. Although both educators understood the details of advisories, in their educational activities they primarily emphasized the need to reduce overall intake of fish that were under advisory.

#### 3.2.3. Core environmental health concept: Health effects

Fewer participants (*n* = 7; 29%) discussed potential health effects/risks of consuming contaminants in fish, though several mentioned addressing risk to increase relevance of the content. A nonprofit program manager (female, 14 yrs. exp.) reported addressing health effects to stimulate anglers’ interest:

Connecting personal health to environment is. . .meaningful connection . . . when people start to think about their own health or the health of their children, they tend to perk up and pay more attention.

An extension educator (female, 14 yrs. exp.) suggested referring to local health impacts:

Whatever number of people had mercury toxicity . . . this isn’t some New York Times report; this happens here . . . to your neighbors. This isn’t just a somewhere-else problem. Without being alarmist . . . this happens, so take it seriously.

Some participants (*n* = 7; 29%) highlighted the importance of using qualifiers to address questions about fish safety. A wildlife agency educator (male, 3 yrs. exp.) said, ‘to the best of our knowledge, this is what we know you shouldn’t eat . . . we’re not sure about the rest’, which was consistent with the educational materials of the health agency. Some nonprofit educators assumed a more cautious stance in the face of data gaps, going beyond the limits identified in state health agency materials, saying, ‘I personally wouldn’t eat fish out of [name of river]’ (male, 3 yrs. exp.).

#### 3.2.4. Core environmental health concept: Vulnerable populations

Seventeen participants (70%) talked about at least one vulnerable population. A few highlighted the challenge of conveying FCA information to male anglers, given the advisories’ greater relevance to women and children. One nonprofit educator reported saying ‘pay more attention if you’re feeding this to your children or your wife is pregnant’. Similarly, a wildlife agency educator (male, 3 yrs. exp.) said:

I joke with older males. . .there’s not much it can do to you. It’s pretty much the women of childbearing age and kids that are the main issue . . . it may make you a little slower thinking; but that’s gonna happen anyway.

#### 3.2.5. Teaching efficacy beliefs

**Figure 2.**
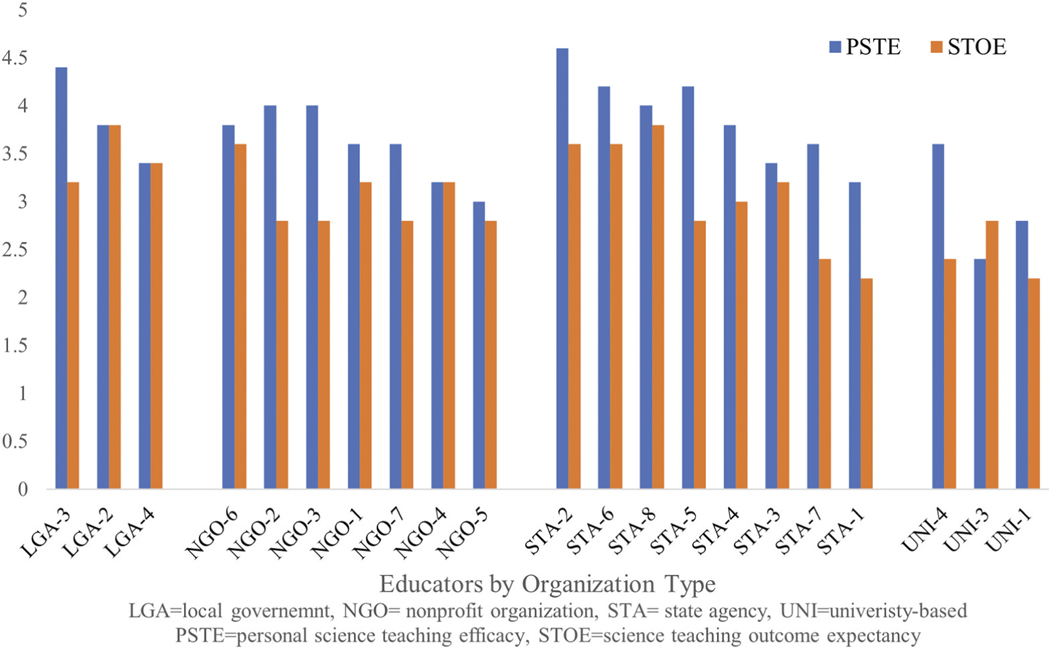
Science teaching efficacy Belief scores among informal educators in different organization types (*n*=22).

Twenty-one participants (88%) completed the modified STEBI. Overall, they indicated higher personal science teaching efficacy (i.e. belief in their ability to teach; average 3.6 out of 5) than outcome expectancy (i.e. resulting learning; average 3.0 out of 5); and this difference was statistically significant (p < 0.00003) (Figure 2). Thus, participants were more confident in their teaching abilities than they were that education about FCAs would have an impact on anglers’ consumption behaviors. They mostly reported feeling efficacious with respect to content knowledge. Twenty participants (95%) agreed or strongly agreed that they understood the related science concepts well enough to be effective in educating anglers, and 18 (86%) agreed or strongly agreed that they typically were able to answer anglers’ questions about the potential harms of eating contaminated fish.

When asked, ‘How do you know when anglers understand?’, a common response (*n* = 16; 68%) was for participants to describe anglers ceasing to ask questions. A health agency educator (male, 3 years of experience (yrs. exp.)) said, ‘Normally it reaches a point where they’re stopping asking questions. . .or they’ve gone into the territory of just asking questions for interest rather than understanding the situation’. Six participants (25%) reported relying on visual cues (such as head nodding or ‘eyes glazing over’), and six reported using pre/post quizzes to assess understanding, as required by their organizations or funders. Almost 40% of participants (*n* = 9) reported that they did not know whether anglers understood the information they provided.

When asked ‘How likely is it that people would take action based on the FCA information you share?’, 16 participants (68%) indicated that they believed at least some anglers acted on the information they provided. Responses ranged from highly likely (*n* = 5) (e.g. ‘probably 90–95 percent of the people act upon the information I give them’) to unlikely (*n* = 5) (e.g. ‘[this information] wouldn’t stop me from eating fish’). Several (*n* = 4) indicated that anglers’ assessment of their organizational credibility likely influenced whether they followed the FCA.

#### 3.2.6. Health beliefs

In reflecting on factors that might influence anglers’ behavior in response to FCA education, participants referenced Health Belief Model constructs, including perceived barriers and benefits to following FCA guidance and the severity of potential health effects.

##### 3.2.6.1. Perceived barriers.

**Figure 3.**
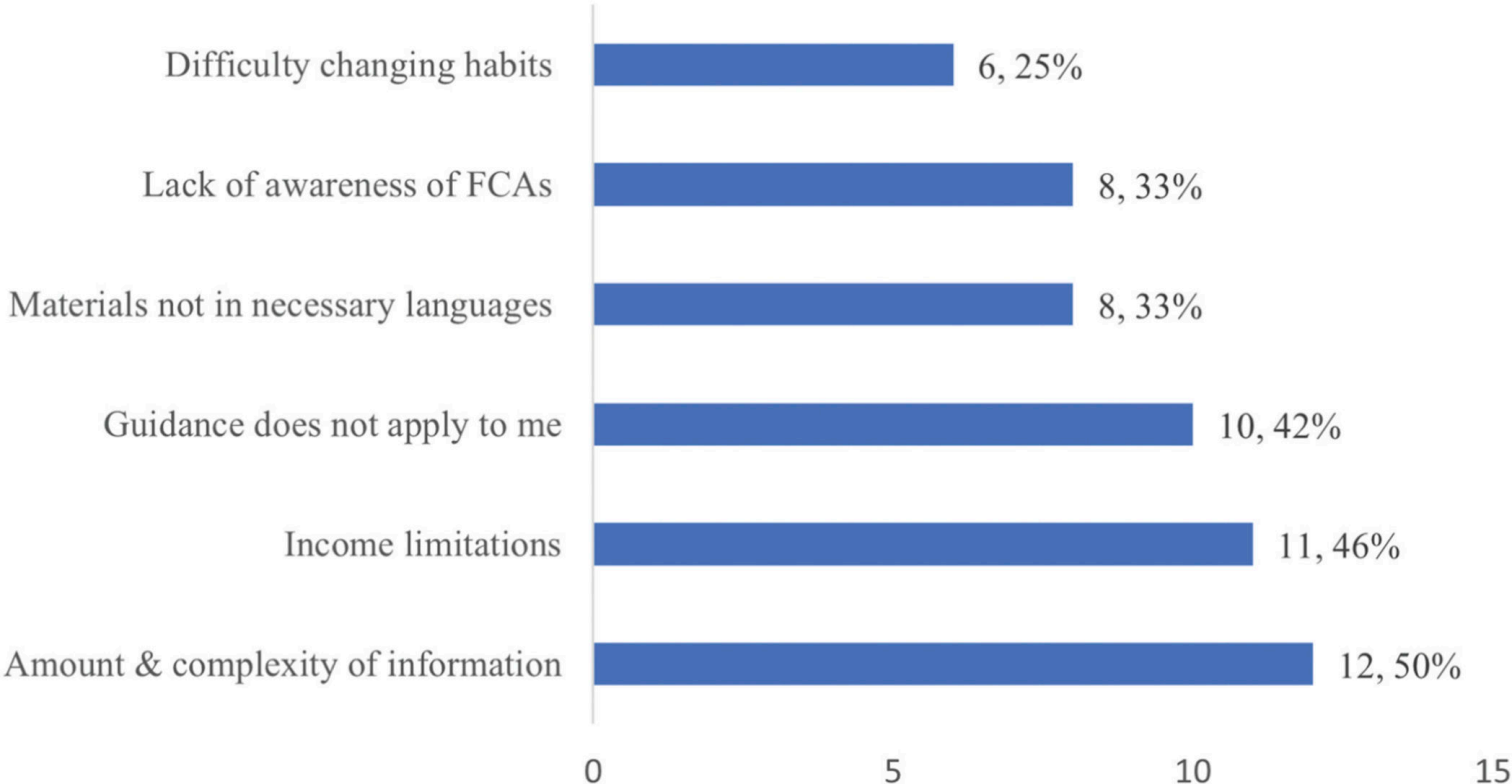
Participant perceptions of common barriers experienced by angles related to following FCA guidance (*N*=24).

Participants identified an average of five barriers (Figure 3) to following advisory guidance; a commonly cited barrier (*n* = 12; 50%) was the amount and complexity of information included in FCAs. A nonprofit educator (male, 7 yrs. exp.) highlighted additional information challenges:

You could go to an obscure website and make about four clicks and finally find it [FCA information] buried at the back end. . .if you were fairly computer literate, had a computer, could read English, and had the patience.

Eleven participants (46%) identified income as a barrier in deciding whether to consume fish under advisory. A nonprofit educator (male, 3 yrs. exp.) underscored the effects of food insecurity saying, ‘If I’m hungry, I’m gonna eat; and I’ll worry about tomorrow tomorrow’. Another nonprofit educator noted that people with less wealth might be less able to travel and access water bodies that were *not* under advisory.

Ten participants (42%) identified lack of perceived relevance as a barrier, based on anglers’ lived experience. A wildlife educator (male, 8 yrs. exp.) reported hearing comments like, “What difference does it make? My granddaddy [fished here and] lived ‘til he was 97”. He noted frequency of consumption as well, saying that anglers told him, ‘I never eat more than two meals [of this fish] a week’. Comments from several participants underscored their impression that most anglers (including vulnerable populations) were not eating enough fish for contaminants to be harmful.

Some participants (*n* = 8; 33%) reported a lack of multilingual educational materials for speakers of Spanish and Asian languages. For populations with fish/mollusks as dietary staples and families who passed down fishing practices through generations, cultural barriers were mentioned as possibly limiting individuals’ receptivity to FCA education.

##### 3.2.6.2. Perceived benefits.

Participants were less able to identify potential benefits to anglers of following FCA guidance, with only 13 (54%) identifying any benefits. Health benefits were mentioned by 10 participants (42%), such as ‘somebody’s health is better protected that day’. A wildlife agency educator (male, 3 yrs. exp.) addressed benefits to vulnerable populations, saying, ‘[Y]ou want your children to be as developmentally healthy as possible and here’s a way you can do that’. Some participants (*n* = 7; 29%) mentioned the potential for anglers to share FCA information with their communities. Others (*n* = 5; 21%) identified knowledge gains as a benefit, suggesting that increased knowledge of risks could enhance anglers’ decision making.

Overall, participants ranked the perceived severity of the health effects of eating contaminated fish as a 6 (mean) on a 10-point scale. The severity for vulnerable populations also was rated a 6 (mean), and their vulnerability as a 7 (mean).

## 4. Discussion

In this study, most educator-angler interactions around FCAs could be characterized as everyday science learning, with anglers encountering scientific content spontaneously and opportunistically (NRC, 2009). Although some participants addressed FCAs in their educational activities, none identified FCA education as a primary function of their jobs. Those who addressed FCAs regularly resembled park naturalists in that they engaged multiple learners in short-duration interactions in natural environments (Taylor & Caldarelli, 2004). Some also addressed FCAs briefly during longer educational interventions focused on other topics. The educators who engaged the highest numbers of anglers annually (state wildlife agency personnel) rarely addressed FCAs in their educational programming and were least conversant in environmental health topics, limiting opportunities for anglers and the public to learn about FCAs from them.

Overall, study participants’ environmental health knowledge could be characterized as incomplete. For instance, many were aware of common contaminants in fish and their potential to harm health; yet fewer could identify potential health outcomes of these exposures and the most vulnerable populations. This finding is consistent with prior FCA research conducted with anglers (Gray et al., 2020; LePrevost et al., 2013; Tan et al., 2011) and may hinder educators’ ability to educate anglers in conversation about potential health risks of consuming contaminated fish. A subset of participants referred to ecosystem concepts in conversations with anglers, using these terms to explain FCAs in what they believed were more accessible or relatable terms, although it is not clear whether anglers would be familiar with the terms (Lauber et al., 2017)

**Figure 4.**
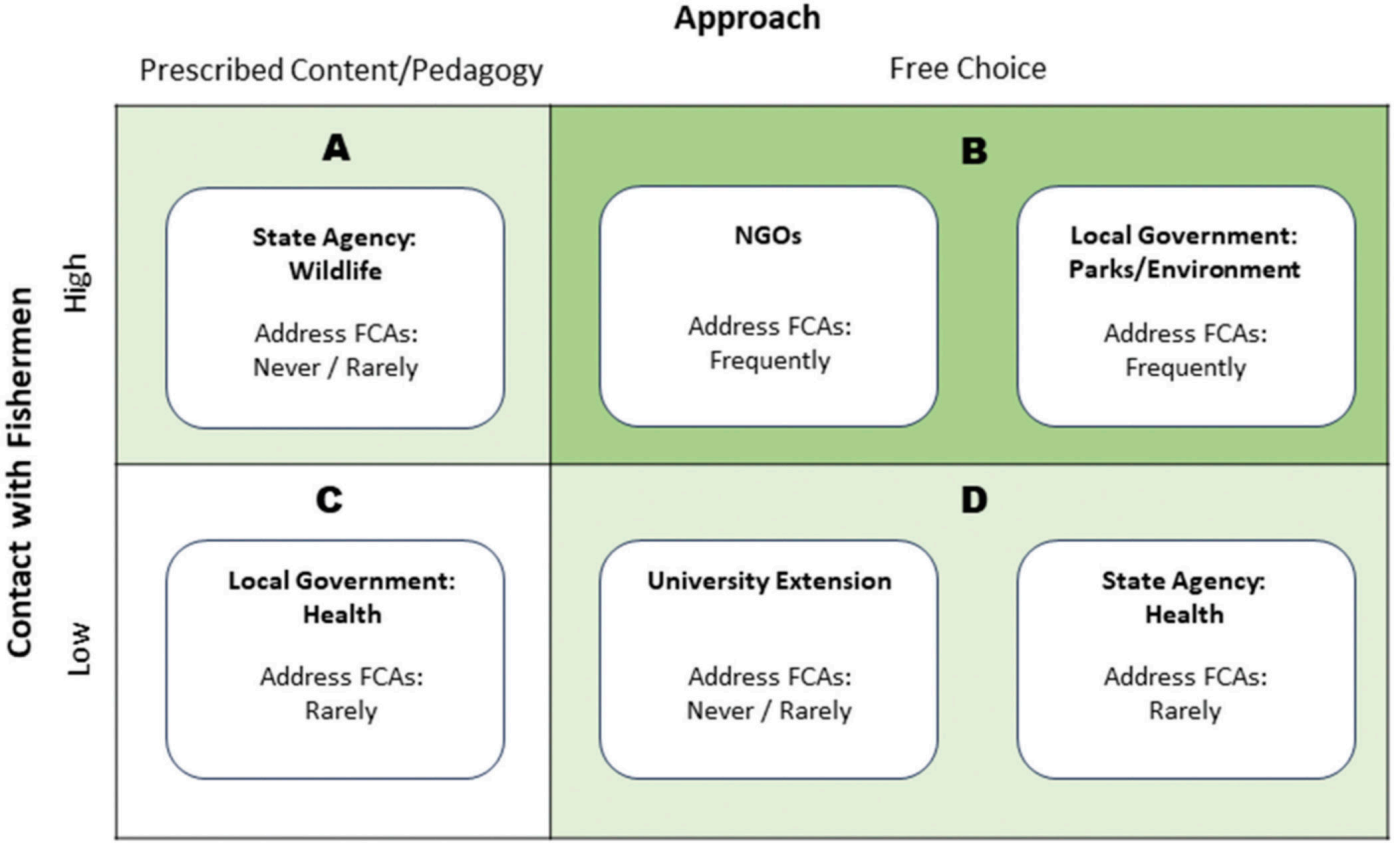
Overview of educational approaches by organization type.

Participant comments about their teaching beliefs provided insight into two additional limitations on FCA education (see Figure 4): (1) educators’ lack of freedom to design educational interventions and (2) limited interactions with anglers. Participants in health departments and wildlife agencies described being required to use established curricula, an institutional constraint that was previously documented with pesticide educators (LePrevost et al., 2013). Health department educators reported the least freedom to decide what information to share, relying on scripts provided by their departments; they also had relatively limited contact with anglers (Figure 4, quadrant C). Wildlife educators, who were housed in the state agency that sells fishing licenses, also described relying on prescribed curricula, though they reported extensive contact with anglers. Notably, several participants recommended that wildlife agency educators could play a greater role in FCA education, due in part to their high level of contact with anglers. This recommendation was consistent with prior research that identified conservation officers as highly credible resources on advisories (Jardine, 2003). Local government participants based in environmental and recreation departments and some nonprofit educators reported regular interactions with anglers and a high degree of freedom in deciding what to teach (Figure 4, quadrant B), a combination that led them to conduct more active FCA education than others. In this way, their positions resembled those of other environmental educators, who have described having freedom to choose and create instructional materials (Pratson et al., 2021).

Some participants reported difficulty in assessing whether anglers understood the information they shared, which could undermine their motivation to educate about FCAs (Pratson et al., 2021). Few used formal assessments to gauge understanding; and those who believed that anglers understood them tended to rely primarily on casual interactions, similar to what has been reported in other environmental education contexts (Taylor & Caldarelli, 2004).

Participants’ science teaching efficacy scores were somewhat low, though generally in the same range as have been reported for other informal educators (LePrevost et al., 2013). These beliefs also could reduce the likelihood that participants would seek out opportunities to educate about FCAs compared to educators with higher science teaching self-efficacy (Quaranta & Spencer, 2015).

In terms of health beliefs, all participants identified barriers that anglers face in following FCA guidance, with some (e.g. income limitations, cultural barriers) being difficult to overcome. Fewer participants identified benefits of following FCAs; and those who did tended to use vague language. Most participants did not perceive the potential health threats to be particularly high. Research has found that this combination of health beliefs—few perceived benefits and lack of perceived threat—undermines the likelihood of an individual taking protective actions or an educator encouraging such actions (Orji et al., 2012; Quaranta & Spencer, 2015).

Given the exploratory nature of this study, the ability to generalize findings is limited. The study sample was small and relatively homogenous, despite involving FCA educators from across the state. A larger and more diverse sample, including educators from other states and regions, could identify additional opportunities and constraints regarding FCA education.

## 5. Conclusions and recommendations

This study addressed a gap in the literature by operationalizing environmental health literacy to address teaching efficacy and health beliefs in addition to knowledge acquisition (Gray & Lindsey, 2019). This study also provided insight into a topic that has not previously been reported in the literature: how informal educators share information with anglers about fish consumption advisories (FCAs) and how their educational activities may be influenced by a combination of knowledge, beliefs, and institutional constraints. Some participants regularly interacted with anglers through planned programming, but few addressed fish consumption advisories in these programs. In contrast, educators who regularly addressed FCAs primarily engaged with anglers in unplanned interactions along waterways, reaching small numbers. In this study, local government environmental and recreation staff and educators working for environmental nonprofits were best positioned to incorporate FCA information into ongoing programming. Wildlife agency educators were positioned to do so but needed organizational support.

As a group, participants demonstrated varied environmental health knowledge relevant to FCAs, and those who interacted with the largest numbers of anglers tended to be less familiar with environmental health concepts. Even educators with comparatively robust knowledge were less aware of potential health effects of common contaminants and at-risk populations, meaning they were not well-positioned to develop tailored education. Additionally, many participants reported teaching efficacy and health beliefs that were inconsistent with encouraging anglers to follow FCA guidance. Thus, opportunities were limited for anglers to learn about advisories from participating educators.

These findings suggest opportunities to provide targeted professional development (PD) that highlights (a) environmental health concepts relevant to FCA education, including risk communication, and (b) skills development relevant to informal education. Such PD could improve educators’ content knowledge and teaching efficacy beliefs associated with FCAs, better preparing them to take advantage of anglers’ interest in learning about fishing topics. Additionally, in states where advisory development and implementation span multiple agencies, leadership should be advised of the ways that institutional constraints on curriculum choices can hinder effective environmental health education. Stakeholder dialogue or other communication with agency leadership could highlight opportunities for improved coordination and more effective education about environmental health risks. Concurrently, informal educators who regularly interact with anglers could be encouraged and supported to share FCA information, especially with vulnerable populations. Finally, research into the health beliefs and knowledge of anglers could assist in identifying any misalignment with informal educators’ knowledge and beliefs and clarify opportunities to improve education about fish consumption advisories.

## Figures and Tables

**Table 1.** Overview of constructs measured and data collection instruments

Construct	Data Sources	Instruments
Environmental health knowledge	Interview transcripts	Semi-structured interview guide, including questions about position, experience/training, concepts shared with anglers, how to assess safety for consumption, FCA information sources
	FCA knowledge and beliefs survey	8-item survey, including 5 questions on contaminants, health effects, vulnerable populations, local FCAs, and regulatory agencies
Teaching efficacy beliefs	Interview transcripts	Semi-structured interview guide, including modified questions from Teacher Beliefs Interview (TBI, Luft and Roehrig (2007)) related to decisions about what to teach
	Teaching efficacy survey	Modified Science Teaching Efficacy Belief Instrument (STEBI, Riggs and Enochs (1990))
Health beliefs	Interview transcripts	Semi-structured interview guide, including questions about concepts shared with anglers, likelihood of anglers acting on FCAs
	FCA knowledge and beliefs survey	8-item survey, including 3 questions on HBM constructs (perceived severity and susceptibility)

**Table 2.** Correct responses to FCA knowledge questions

	Number Correct	Percent Correct
**Contaminants Included in FCAs**		
Mercury	19	86%
PCBs	14	64%
Dioxins	5	23%
**Health Effects**		
Neurological effects	10	46%
Cancer	7	32%
**Vulnerable Populations**		
Children	11	50%
Subsistence fishermen	8	36%
Pregnant/breastfeeding women	7	32%
**Agency that Creates FCAs**		
NCDHHS	10	46%

## Data Availability

Participants did not consent to having their interviews made available.

## Footnotes

1. Although route of exposure also is a core environmental health concept, it was not included because fish consumption (or ingestion) was the focus of these interviews. As a result, the interviewer posed questions that addressed this route of exposure in all interviews, making it difficult to determine how participants would have introduced this concept on their own.
